# “I was determined to breastfeed, and I always found a solution”: successful experiences of exclusive breastfeeding among Chinese mothers in Ireland

**DOI:** 10.1186/s13006-020-00292-x

**Published:** 2020-05-20

**Authors:** Qianling Zhou, Haoyue Chen, Katherine M. Younger, Tanya M. Cassidy, John M. Kearney

**Affiliations:** 1 Department of Maternal and Child Health, School of Public Health, Peking University, Beijing, China; 2 School of Biological and Health Sciences, Technological University Dublin, Kevin Street, Dublin 8, Ireland; 3 School of Nursing, Psychotherapy and Community Health, Dublin City University, Dublin 9, Ireland

**Keywords:** Exclusive breastfeeding, Chinese, Immigrant, Ireland, In-depth interviews

## Abstract

**Background:**

The prevalence of exclusive breastfeeding for at least 4 months was previously found to be very low among Chinese immigrants in Ireland, at 5.8% (Zhou et al., Front Public Health 6:351, 2018). This study investigates the successful experiences of Chinese mothers living in Ireland who exclusively breastfeed for between four and 6 months.

**Methods:**

Participants were recruited from the sample of the Ireland Chinese Mother Survey. Qualitative in-depth interviews were conducted with fourteen participants in their homes or public places.

**Results:**

A content analysis revealed that various factors contributed to a successful experience of exclusive breastfeeding among the group of Chinese immigrant mothers, including strong self-determination; appropriate physical conditions; awareness of the benefits of exclusive breastfeeding; a lack of time constraints; and family, professional and policy support. The barriers that the mothers faced included the difficulty of balancing breastfeeding and employment, infant health issues, language barriers, an inability to consume the traditional Chinese postpartum diet and a lack of public breastfeeding facilities. Measures taken to overcome these barriers included seeking family support, resting during the lactation period, and pumping breast milk to feed from a bottle when outside the home.

**Conclusions:**

This study highlights unique factors affecting exclusive breastfeeding among Chinese mothers in Ireland, which may be useful to health care professionals working with Chinese immigrant women internationally.

## Background

Breastfeeding has short-term and long-term health benefits for infants and mothers. Numerous studies have shown that breastfeeding has protective effects against infectious diseases in infancy [1], obesity in childhood and adulthood [2], and breast [3] and ovarian cancer [4] for mothers. Breastfeeding also improves mother-child bonding and reduces the cost of medical care for society [5]. Exclusive breastfeeding is defined as feeding an infant with human breast milk only with provision of oral rehydration solutions, drops, and syrups if needed [6]. There is growing evidence that exclusive breastfeeding for 6 months is the optimal way to feed infants [7, 8]. Early introduction of solids before 4 months is associated with an increased risk of childhood obesity [9]. Premature weaning has been associated with a range of chronic diseases in adulthood [10]. The World Health Organization (WHO) recommends exclusive breastfeeding for the first 6 months of life, with the introduction of solid foods thereafter and continued breastfeeding for up to 2 years of age or beyond. The introduction of solids before 6 months is not recommended [11]. According to the United Nations International Children’s Emergency Fund, a child who is exclusively breastfed is 14 times less likely than a non-breastfed child to die during the first 6 months of life [12]. The WHO has set a global target of exclusive breastfeeding for 50% of the world’s infants at 6 months of age by the year 2025 [13].

Fewer than 40% of infants aged under 6 months were exclusively breastfed globally between 2007 and 2014 [11]. In China, the national nutrition report demonstrated that although the prevalence of ever breastfeeding was 79.6%, only 20.8% of infants were exclusively breastfed for 6 months in 2013 [14]. According to the World Health Statistics 2013, the exclusive breastfeeding rate at 6 months in Ireland was 15%, compared with the average rate of 25% in Europe [15].

Migration to another country has an impact on breastfeeding practices [16, 17]. Chinese immigrants, who constitute 3% of non-nationals represent one of the largest ethnic groups in Ireland [18]. The Chinese immigrant population contributed around 17% to the increase in the non-Irish population between 2011 and 2016 [19]. More than 85% of Chinese women living in Ireland are of child-bearing age (20–39 years old) [18]. The Ireland Chinese Mother Survey investigated the breastfeeding practices of Chinese mothers who gave birth in Ireland between 2008 and 2009, and showed that these mothers Ireland breastfed for a shorter duration than mothers who gave birth in China [16, 20]. Cultural conflict, socioeconomic problems and reliance on infant formula contributed to the early cessation of breastfeeding among these Chinese mothers [16, 20]. Only 5.8% of Chinese mothers exclusively breastfed their babies for at least 4 months [16, 20].

Barriers to breastfeeding among immigrants have been reported in the literature [21, 22], however, specific solutions remain undocumented. The aim of this study was to explore the experiences of Chinese immigrant mothers who exclusively breastfed for four to 6 months. Using a qualitative approach, the specific objectives of the study were to 1) identify factors contributing to the success of exclusive breastfeeding; and 2) find solutions to the barriers impeding exclusive breastfeeding among immigrant Chinese mothers in Ireland.

## Methods

### Study design

Detailed, semi-structured individual interviews were conducted to gain an in-depth understanding of successful exclusive breastfeeding experiences among Chinese immigrant mothers in Ireland as this methodology was suggested as appropriate for immigrant populations [23, 24]. Interviews were conducted between December 2009 and February 2010. The study was approved by the Research Ethics Committee of the Dublin Institute of Technology. The purpose and confidentiality of the study were explained to the participants, and written consent was obtained before each interview.

### Participants

The participants were selected purposively from the respondents to the Ireland Chinese Mother Survey [16, 20]. Eligible participants were born in China, had lived in Ireland for more than 6 months, gave birth in Ireland, breastfed their child (ren) for at least 6 months, and exclusively breastfed for four to 6 months. All mothers who met the inclusion criteria were invited via telephone calls to participate in the study. Owing to time constraints, two mothers declined to participate. A total of 14 individual interviews were conducted.

### Setting and data collection

Interviews were conducted in the participants’ houses or public coffee shops or wherever was convenient for the participants. Author, QZ, a female doctoral student in public health nutrition conducted the interviews. She speaks Chinese (both Mandarin and Cantonese) as her mother languages and received intensive training in qualitative research. Author, TMC is an anthropologist with qualitative research experience in lactation. TMC guided QZ in the study development, implementation and analyses. All interviews were conducted in Mandarin according to the participants’ preferences, lasted between 45 and 125 min, and were audio-recorded with the participant’s permission. Immediately after each interview, QZ wrote field notes on her observations, memos and unverifiable information. Data saturation was achieved during the final interview [25] and the interview transcripts were returned to the participants for confirmation before the coding began.

A semi-structured interview guide was developed by the research panel (authors QZ, TMC, KMY, and JMK) and reviewed by several Chinese immigrant mothers who gave birth in Ireland but did participate in the study. The guide started with an open-ended question on mothers’ personal experiences of childbirth and infant feeding in Ireland. Mothers were prompted to share how they initiated and sustained exclusive breastfeeding. The main open-ended interview questions were as follows and adjustments to specific questions were made during interview according to the interview context [26].

1. How did you manage to exclusively breastfeed for four to 6 months?
2. Did you encounter any barriers to exclusive breastfeeding? If yes, please describe the barriers and how you overcame them.
3. Have you ever thought about giving up breastfeeding? If yes, please describe how you finally continued to exclusively breastfeed.

### Data analysis

All interviews were transcribed verbatim in Mandarin by QZ, and any personal details were de-identified. The content analysis was conducted by HC and QZ following the guidelines recommended by Morse and Field [27]. Open coding was conducted by reading the transcripts sentence by sentence. Initial codes were generated and then organized into categories and integrated with the assistance of a coding tree. The content was then reviewed and refined, first at the level of the coded data and then at the level of the categories. All interviews were rigorously coded, verified and agreed upon through discussion between QZ and HC. Field notes were reviewed against the transcripts during this process. The results were then translated and interpreted in English.

## Results

### Sample characteristics

Fourteen mothers participated in this study; eight had only one child at the time they were interviewed and six had two children (one with twins). The mothers were between 24 and 54 years old (mean age, 34 years) and at the time of the study had lived in Ireland from between three to 18 years (mean duration: 9 years). The mothers generally had a high level of education; the majority had reached third-level education and some had attended secondary or training schools. Over half were stay-at-home mothers or had part-time non-professional jobs, while the remainder were self-employed or had professional jobs.

### Mothers’ successful experiences with exclusive breastfeeding

A thematic analysis of the data revealed two themes: *favourable factors for exclusive breastfeeding,* and *difficulties with exclusive breastfeeding and specific solutions*.

### Maternal and infant factors

Maternal factors, including physical and psychological factors, awareness of the benefits of breastfeeding, and a lack of time constraints, were indicated to be important for exclusive breastfeeding.

Most mothers perceived that their breast milk was sufficient and of good quality, and that their nipples were a suitable size for infant sucking. “*My nipples are big, so they are suitable for my baby to suck. In comparison, my friend’s nipple size is not that suitable for suckling, and she gave up breastfeeding.*” (P7).

The mothers believed that self-determination was of foremost importance. They showed a strong will to breastfeed and obtained relevant knowledge judiciously rather than blindly relying on others. “*I was determined to breastfeed, and I always found a solution (any time I came across breastfeeding problems)*.” (P6).

The mothers also realized that “breast is best”. First, they believed that exclusive breastfeeding increased infants’ immunity, improved infants’ responses to satiety, prevented excessive milk consumption and improved the function of the respiratory system. “*Breast milk can improve babies’ health, especially their resistance*” (P1). “*Babies fed by formula wouldn’t feel full until they stop feeding, while those who are breastfed would stop sucking when they are not hungry*” (P9). Second, they believed that exclusive breastfeeding helped restore their normal weight and reduced maternal risk of breast disease. They also suggested that intimacy and bonding between mothers and babies increased during lactation and might also relieve maternal depression. “*Breastfeeding prevents mothers from breast disease*” (P2). Third, regarding benefits for society, the mothers mentioned that exclusive breastfeeding enhanced the overall physical health of babies, reduced infant morbidity and social health risks, and relieved the social burden. In terms of the economy and environmental protection, they considered exclusive breastfeeding reduced the consumption of infant formula which decreased the economic burden, saved resources and protected the environment. “*Compared with formula, breastfeeding is convenient, healthy and environmentally friendly, and generates many benefits for the government*” (P11). Finally, they reported that the tedious steps of feeding formula could be avoided through breastfeeding. Additionally, most mothers were housewives or had part-time non-professional jobs, which left them sufficient time for breastfeeding. “*I had enough time to breastfeed because I didn’t have a job*” (P5). For those who had professional jobs, their maternity leave was as long as 6 months.

Some mothers reflected that they had to continue breastfeeding because of their babies’ inability to drink from a bottle and refusal of infant formula.

“*I tried weaning at six months but failed because the textures of a rubber nipple and a human nipple were completely different. I had tried almost all kinds of bottles, but he refused to drink even when he was hungry all day.*” (P10)

### Cultural influences

Chinese breastfeeding culture was reported to have influenced the decision to initiate breastfeeding and to persist with exclusive breastfeeding for 6 months. When the mothers were asked, “*What is the most important thing that made you succeed in sticking to exclusive breastfeeding for six months*” they often answered, “*Chinese traditional culture*” and indicated that they wanted to breastfeed for as long as their mothers had (P5).

### Family support and peer influence

Ten of the 14 mothers had family members arrive from China to provide childcare. All said they received support from their husbands, parents, or parents-in-law. They also mentioned that encouragement from family members helped them overcome difficulties (e.g concern about their breasts sagging and darkening after breastfeeding for a long period) and to continue exclusive breastfeeding. Husbands’ high appreciation for the practice was vitally important. In addition, most mothers appreciated the physical support provided by family members. They also believed that their ability to rest, engage in normal daily activities and consume Chinese postpartum soups, boosted the quantity and quality of their breast milk:

“*I had good rest, as my parents came here helping to look after the two babies.*” (P3)

“*My husband came back every night and cooked fish soup for me*.” (P8)

The mothers were encouraged by their peers’ successful experiences of breastfeeding. Community breastfeeding groups, where mothers exchanged breastfeeding experiences and encouraged optimal feeding practices, provided reliable evidence about the feasibility of exclusive breastfeeding.

“*One of my good friends breastfed her three babies for nine months. I initiated breastfeeding to a large degree, because of her successful experience.*” (P10)

### Employer, policy and health professional support

Some mothers mentioned that the flexibility of work, the establishment of nurseries near their workplaces and the understanding of employers contributed greatly to continuing to breastfeed after returning to work. Others indicated that lactation policy ensured their right to breastfeed at work.

“*My work was flexible... it was easier (for me) to have the time and the right place for breastfeeding than (it was for) other mothers.... My boss also supported (me) a lot … . What’s more, the nursery was close to my workplace so that I could see my baby during the break.*” (P13)

The mothers mentioned that Irish hospitals provided breastfeeding facilities, consultations, and education. Community health centres offered health check-ups and lactation support on a regular basis. Public nurses and midwives helped them solve lactation problems (e.g. relieving breast pain) and provided relevant infant feeding information during their regular postpartum home visits.

“*The encouragement given by the doctor was also very helpful. During the first three days, I didn’t produce breast milk, which made me dispirited. But the doctor encouraged me: ‘You must stick to it. There will be (enough milk).*’” (P3)

### Difficulties with exclusive breastfeeding and specific solutions

Difficulties encountered by the mothers during breastfeeding included common barriers, problems with their infants and obstacles faced as immigrants (see Table 1). Relevant solutions for these difficulties included reading books and brochures from materiality hospitals, attending prenatal maternal classes, consulting health care professionals, surfing the Internet, participating in community breastfeeding groups and learning from other people.

**Table 1:** Barriers to exclusive breastfeeding encountered by Chinese mothers and relevant solutions

Difficulties			Solutions
Maternal barriers	Breast milk quantity and quality	Insufficient breast milk	• Promote frequent sucking • Maintain a balanced diet and eat plenty of soup • Rest well and maintain a good mood • Feed for shorter durations and more times a day
		Excessive production of breast milk	• Pump milk before breastfeeding
		Decreased quality of breast milk as child aged	• Maintain physical health
		Decreased quality of breast milk during illness	• Continue breastfeeding, as antibodies from the mother could be passed to the baby through breastfeeding
	Breast problems	Presence of lumps during initial milk production	• Massage the breast with a hot towel
		Engorgement	• Use a breast pump frequently
		Baby teething	• Communicate with baby and pat him or her gently
		Cracked nipples	• Use an edible ointment • Stimulate the nipples with rough towels before giving birth
		Blocked breast ducts	• Continue breastfeeding • Apply heat and cold to the breast
		Breast inflammation	• Massage and clean the breast
	Concerns about breast shape	Breast sagging	• Wear a bra that prevents sagging
		Breast asymmetry	• Breastfeed from both breasts
	Difficulties with lactation after returning to work	Inability to take care of the baby	• Pump milk in advance • Put the baby in a crèche • Adjust working hours
		Decreased breast milk production	• Increase workload gradually rather than having full load at the beginning
	Inability to balance lactation and other daily activities	Inability to balance lactation and work	• Manage time reasonably • Seek family support
		Inconsistent sleep schedule between mother and infant	• Adjust the baby’s schedule • Adapt to the baby’s schedule • Use a breast pump • Seek family support
		Inability to balance lactation and childcare	• Seek family support
	Other problems	Dietary and behaviour restrictions	• To be determined
		Consumption of medication	• Suspend breastfeeding while taking medication • Choose medication that can be taken during lactation • Do not take medication
		Maternal depression	• Self-regulation and self-control
Infant barriers	Breast milk jaundice		• Continue breastfeeding, increase the frequency of breastfeeding • Promote infant secretion • Increase infant sun exposure
	Severe diarrhoea		• Consume a maternal diet low in fat and sugar
	Inability to latch		• Rub around the baby’s lips
Cultural barriers and social adjustment	Language barrier		• Employ Chinese health care professionals
	Inability to consume a Chinese postpartum diet		• N/A
	Odd looks or negative comments from the public		• Ignore negative attitudes or behaviour • Use a cloth to cover the breast
	Lack of breastfeeding facilities in public		• Pump milk before going out • Reduce the frequency of going out, avoid going out, or choose places near home • Choose places that have breastfeeding facilities • Use hidden places to breastfeed (e.g.*,* toilets, cars)

### Maternal and infant barrier to breastfeeding

Maternal barriers to breastfeeding included breast milk quantity and quality, breast problems, concerns about breast shape, difficulties with lactation after returning to work, inability to balance lactation and other daily activities, diet and medication.

Some mothers indicated that they had insufficient breast milk. To solve this problem, they began breastfeeding their babies immediately after birth, breastfed for shorter intervals and more times a day, maintained a balanced diet, frequently ate soup (Chinese postpartum diet), ensured they had adequate rest and tried to maintain a good mood. In contrast, those who produced too much breast milk, which could make babies choke easily, pumped some breast milk before feeding. In addition, mothers worried that the quality of their breast milk decreased as their babies aged. To avoid this problem, they monitored their bodies by observing the colour of their breast milk and making appropriate adjustments to maintain their physical health. Many mothers were concerned that the quality of their breast milk might decrease because of maternal illness (e.g. cold, fever). Some mothers continued breastfeeding while ill, as they learned from medical articles that antibodies from the mother could be passed to babies through lactation:

“*As my babies grew up, I felt that my breast milk might fail to meet their nutritional needs . . . So, after they were six months of age, I observed the colour of my breast milk and tried to maintain good health to continue breastfeeding.*” (P4)

Most mothers experienced severe pain and discomfort during lactation due to the secretion of breast milk, breast engorgement, baby teething and cracked nipples. To remove lumps during initial milk production, they massaged the breast with a hot towel. To relieve pain caused by engorgement they used breast pumps frequently to remove milk. During baby teething, based on their understanding of why babies bite, the mothers communicated with their babies by patting them gently on the cheek. To prevent cracked nipples, they stimulated their nipples with rough towels before giving birth and applied edible ointment to relieve cracking. Additionally, some mothers experienced blocked ducts and breast inflammation. To overcome these difficulties they continued breastfeeding, applied hot and cold packs to the breast in combination with regular breast massage, and ensured their nipples were kept clean.

“*When I initially secreted breast milk, I was very uncomfortable, because my whole breasts were hard and full of lumps as big as cocoons … It hurt so much … My husband massaged my breast with a hot towel.”* (P2)

“*I patted him gently on the mouth and told him not to bite me. Since then, he never bit again.*” (P11)

“*The public nurse prevented me from stopping breastfeeding... She said that the baby’s sucking and a hot pack with a towel could relieve the blockage of breast ducts.*” (P11)

Mothers were also concerned about breast sagging and asymmetry. They mentioned that wearing a breastfeeding bra was helpful. To avoid and relieve breast asymmetry, they breastfed from both breasts.

“*After weaning my first child, the shapes of my breasts were different . . . so I fed my second baby with the smaller one, more times.*” (P9)

Mothers with short maternity leave had to return to work early during lactation, leaving no time for baby care. To solve this difficulty, they pumped milk in advance, put their babies in crèches and adjusted their working hours. Some mothers reported that returning to work full time led to a decrease in breast milk production. To solve this issue, they increased their workload gradually.

“*When he was three months old, I sent him to the crèche and pumped breast milk into a bottle, but he refused to drink a bottle, so I breastfed in the crèche once every three hours … . Three months later, the staff … could feed him with spoon, so I breastfed during lunch time.*” *(P13)*

“*I adjusted the working hours with my husband. My husband brought our baby to my workplace twice a day so that I could continue breastfeeding.*” (P7)

Some mothers complained that their inability to balance lactation, work, childcare and sleeping made them feel tired and restricted. To solve these problems, they sought family support and managed their time reasonably. In addition, they used breast pumps, adjusted their babies’ schedules, or adapted to their babies’ schedules.

The mothers complained about dietary and behavioural restrictions during lactation. However, they were determined and showed a strong will for the sake of their babies’ health. “*During lactation, I couldn’t eat too salty or too spicy [food], but I like spicy food very much*. *.*. *Every time I saw a dish of tasteless soup [the traditional postpartum diet], I felt sick*. *.*. *But I still ate it for my babies’ sake*” (P8). Moreover, taking medication while breastfeeding was a major issue. Some suspended breastfeeding while taking medication, some chose medication that could be taken while breastfeeding and others did not take any medication. Finally, some mothers reported maternal depression and irritation. As they realized that maternal depression could result in their babies’ refusal to suck, they tried to calm themselves through self-regulation and self-control.

Some mothers also reported their infants having breast milk jaundice, severe diarrhoea, and failing to successfully latch. Those whose infants had breast milk jaundice, continued breastfeeding and increased the frequency to promote infant urination. Increasing the baby’s exposure to the sun was also cited as a useful method. To relieve severe diarrhoea, the mothers consumed a diet low in fat and sugar, and resolved latching problems through rubbing their nipples around the baby’s lips.

“*My baby had jaundice due to breastfeeding. Chinese doctors advised stopping breastfeeding for one or two weeks, but Irish medical staff advised increasing breastfeeding frequency to promote infant digestion and defecation. I insisted on this and continued to feed her.*” (P6)

“*The nurse taught me to rub [my nipples] around her lips, telling her that the food was coming. Another woman taught me to flatten the nipple and make it smaller. I did what they said, and I succeeded.*” (P14)

### Cultural barriers and social adjustment

The language barrier and inaccessibility of a Chinese postpartum diet were major barriers to breastfeeding among the mothers. They also indicated difficulty understanding medical terminology and strongly recommended employing Chinese health care professionals in Irish hospitals. “*In fact, there was still a language barrier. When I communicated with a health care professional, there were many professional terminologies that troubled me. So, I think maybe more Chinese nurses and midwives would be better.*” (P6).

The mothers reported receiving odd looks or negative comments whenever they breastfed in public. They found ignoring negative attitudes was the best way to deal with the problem and using a cloth to cover the breast while breastfeeding was also helpful. The mothers also complained that in Ireland only limited breastfeeding facilities were available in public places. To solve this problem, they reduced the frequency of breastfeeding, avoided going out and chose places near home or those that had breastfeeding facilities. They pumped milk before going out or breastfed in hidden places (e.g. the toilet or the car).

“*(You should) ignore others’ prejudice. It is largely down to your own opinion on breastfeeding and your self-determination.*” (P1)

“*I placed a scarf on baby’s head when I had to breastfeed in … public. This created a small private space for breastfeeding.*” (P6)

“*I went to familiar places that had special rooms for breastfeeding . . . If I found no place for breastfeeding, I went back to the car . . . I went to places near my house more often.*” (P5)

Additionally, one mother indicated that a health care professional’s comment had a negative impact on breastfeeding: “*When I was still in the hospital, my baby was unable to latch the first two nights after birth. The nurse said that the shape of my nipples was not suitable for feeding. I didn’t understand why she gave me such a negative comment.*” (P1).

## Discussion

This study showed that various factors contributed to Chinese immigrant mothers’ successful experiences of exclusive breastfeeding. Favourable factors included strong self-determination, appropriate physical conditions, awareness of the benefits of exclusive breastfeeding, a lack of time constraints, support from the people around them and policy support. Barriers that Chinese immigrants encountered while breastfeeding included common lactation barriers, difficulties as new immigrants and adjusting to the Irish environment.

The mothers’ experiences demonstrated that self-determination enabled them to overcome the barriers they faced and was important to the success of exclusive breastfeeding. A similar finding has been reported among Hong Kong mothers who breastfed exclusively for 6 months [28]. This study recognized that mothers having determination is one of the ways to persistence in overcoming barriers [28]. Our results suggest a need to develop programmes that are useful in enhancing self-determination to exclusively breastfeed.

Our study participants reflected that their initiation of and adherence to exclusive breastfeeding were shaped by Chinese culture. This finding is consistent with the literature that breastfeeding among Chinese immigrants is influenced by traditional Chinese health beliefs [21, 29–31] and that people with negative attitudes towards breastfeeding are less likely to breastfeed for a long period [21, 29–32].

Our participants reported their infants’ reluctance to accept infant formula as a contributing factor to prolonging breastfeeding. This finding has not been reported in the literature, although numerous studies have reported the introduction of infant formula as one of the causes of weaning among Chinese immigrants [17, 21, 31, 33, 34]. Our mothers explained that the different textures of a rubber nipple and a human nipple led to this result. Studies have shown that pacifier use can result in early weaning as it may be associated with changes in the sucking patterns of infants, demonstrating the possible existence of nipple confusion and its effect on breastfeeding [35, 36]. Therefore, avoiding the use of pacifiers while breastfeeding should be emphasised during prenatal education.

In accordance with the literature [17, 21, 29–34, 37], our study participants emphasized the key role that babies’ fathers and grandparents played in successful exclusive breastfeeding. In addition to family support with childcare and consumption of a Chinese postpartum diet which was considered beneficial to the production of breast milk, our study participants ensured they had sufficient rest. Given the importance of family support, the promotion of and education about exclusive breastfeeding might include not only immigrant mothers but also their family members, especially their husbands. For this ethnic group, husbands were the main source of family support [16]. Thus, policies ensuring husbands’ entitlement to maternity leave should be considered.

The literature suggests that the experiences of peers might make immigrant mothers find breastfeeding difficult [28, 38] and undermine their confidence in breastfeeding [28]. In our study, the participants’ maintenance of exclusive breastfeeding after encountering difficulties was influenced by the successful experiences of their peers and the positive impact of breastfeeding groups. This finding suggests that exclusive breastfeeding could be promoted through sharing experiences and encouraging each other within breastfeeding groups. This finding is consistent with the results of a study of Chinese immigrants in Australia where community groups are venues for finding solutions for feeding problems [21].

The improvement of employment opportunities for immigrants was associated with a shorter duration of exclusive breastfeeding [30, 32]. A study of Canadian Chinese immigrants showed that returning to work was a common reason for the early introduction of infant formula and solids [21]. More than half of the mothers in our study were housewives or part-time workers and had sufficient time for exclusive breastfeeding. The remaining participants (43%) who were self-employed or had a professional job, indicated that the flexibility of work, the location of a crèche near the workplace and empathy from employers was important when continuing exclusive breastfeeding after returning to work. Policy support also ensured the mothers’ right to rest and breastfeed at work.

Our study revealed that Irish health care professionals provided support to Chinese immigrants such as strategies to solve breastfeeding problems, prenatal education and encouragement. A qualitative study [21] showed that Chinese immigrants valued and respected the advice of health care professionals, which allowed them to obtain a better understanding of optimal infant feeding methods. Previous studies [32–34] reported that the more that Chinese women who gave birth in Australia received breastfeeding support from health care professionals, the more likely these mothers were to breastfeed immediately after birth.

Although our study participants successfully breastfed for at least 4 months, they found it difficult to do so. The common barriers reported in our study included difficulties such as blocked breast ducts, breast inflammation, breast engorgement, baby teething, reduced breast milk supply after returning to work, an inability to balance lactation with other daily activities, concerns about breast shape, dietary restrictions, medication, postpartum depression, babies’ inability to latch, jaundice, and diarrhoea.

Previous studies have shown that problems such as nipple soreness and cracked nipples were the most common causes of breastfeeding cessation [39]. Our study participants encountered these problems and found baby teething was a major cause of cracked nipples. The impact of teething on exclusive breastfeeding has not been intensively reported in the literature. A quantitative study might be needed to verify the association between baby teething and the duration of excusive breastfeeding.

Previous studies among Chinese immigrants in Ireland [30] and Australia [33] revealed that although mothers believed breastfeeding could help them to quickly recover their body shape, they agreed that breastfeeding could cause also breast sagging. Similar beliefs were reported by our participants who adopted various solutions to prevent breast sagging and continued breastfeeding. The effectiveness of such solutions might be further explored.

Studies have reported the impact of work on breastfeeding practices among Chinese immigrants [22, 31]. Our participants also pointed out that returning to work made their adherence to exclusive breastfeeding difficult. Some of the mothers pumped breast milk so their babies could be fed by family members. Others put their babies in nurseries near their workplace so they could regularly breastfeed. Some, along with their husbands, adjusted their working hours, others increased their workload slowly to avoid a reduction in their milk supply. Such solutions might be further promoted in education on exclusive breastfeeding.

Consistent with the migration literature [21, 40], language barriers were also reported in the current study. Language barriers can lead to a lack of appropriate infant feeding information [40] and an inability to source adequate health professional support [21]. Consequently, there is a need in Ireland to publish language-specific breastfeeding materials and train Chinese health care professionals.

The attitude towards breastfeeding in public is positive in mainland China [41] due to universal education on breastfeeding, while the attitude has been reported to be negative in Ireland [42]. Our study participants recounted receiving negative comments and reactions from the public whenever they breastfed in public. Such experiences have also been reported in studies with mainland Chinese mothers in Hong Kong [28]. Such a finding suggests a need to improve breastfeeding publicity and education to increase the public’s acceptance of breastfeeding. The literature has seldom reported breastfeeding facilities in public places or solutions for public breastfeeding. Our study revealed that there was a lack of breastfeeding facilities in public in Ireland, causing great inconvenience for breastfeeding mothers. Resolving the issue of a lack of public breastfeeding facilities identified in this study might further promote exclusive breastfeeding.

### Strengths and limitations

This study explored the successful exclusive breastfeeding experiences of fourteen immigrant Chinese mothers living in Ireland. The strength of this study is that little qualitative research has been reported on the barrier to breastfeeding among Chinese mothers in Ireland. Besides, even though some barriers identified in this study have been reported in the literature, this is the first to present potential solutions to each of the barriers identified. However, no interviews were conducted with the immigrants’ husbands or family members, which limits the qualitative results. Further research is required to explore the breastfeeding support experience of the immigrants’ husbands or family members. Additionally, almost all participants included in our study were highly educated. The experience of mothers with relatively low education levels were under-represented. Further research on this group of breastfeeding mothers is warranted.

## Conclusion

This study highlights unique favourable factors and barriers to exclusive breastfeeding among Chinese mothers in Ireland. The important role that self-determination played in overcoming barriers suggests a need to develop programmes to enhance self-determination to support exclusive breastfeeding. The significance of family support highlighted in this study suggests the importance of the involvement of family members, especially husbands. The study also draws attention to the urgent need for consistent training of health care professionals, the provision of multilingual health care professionals, and the inclusion of breastfeeding facilities in public places. Our findings may have implications for Chinese immigrant women internationally.

## Data Availability

The data used and/or analysed during the current study are available from the corresponding author on reasonable request.

## Abbreviations

WHO: World Health Organization
UNICEF: United Nations Children’s Fund
